# Quantifying Preferences and Responsiveness of Marine Zooplankton to Changing Environmental Conditions using Microfluidics

**DOI:** 10.1371/journal.pone.0140553

**Published:** 2015-10-30

**Authors:** Nirupama Ramanathan, Oleg Simakov, Christoph A. Merten, Detlev Arendt

**Affiliations:** 1 European Molecular Biology Laboratory, Heidelberg, Germany; 2 Okinawa Institute of Science and Technology, Okinawa, Japan; GEOMAR: Helmholtz Center for Ocean Research, GERMANY

## Abstract

Global environmental change significantly affects marine species composition. However, analyzing the impact of these changes on marine zooplankton communities was so far mostly limited to assessing lethal doses through mortality assays and hence did not allow a direct assessment of the preferred conditions, or preferendum. Here, we use a microfluidic device to characterize individual behavior of actively swimming zooplankton, and to quantitatively determine their ecological preferendum. For the annelid zooplankton model *Platynereis dumerilii* we observe a broader pH preferendum than for the copepod *Euterpina acutifrons*, and reveal previously unrecognized sub-populations with different pH preferenda. For *Platynereis*, the minimum concentration difference required to elicit a response (responsiveness) is ~1 μM for H^+^ and ~13.7 mM for NaCl. Furthermore, using laser ablations we show that olfactomedin-expressing sensory cells mediate chemical responsiveness in the *Platynereis* foregut. Taken together, our microfluidic approach allows precise assessment and functional understanding of environmental perception on planktonic behaviour.

## Introduction

The accelerating pace of human interferences with marine ecosystems has a significant effect on species composition [1–6]. Understanding the effect of various changes on marine micro-communities has become a recent focus of research [7]. Around 75% of the ocean biomass is composed of microscopic marine plankton, such as phyto- and zoo- plankton [8]. Furthermore, targeted settlement of marine larvae determines the micro- and macrostructure of marine ecosystems [9]. So far it has been technically challenging to monitor the impact of fluctuations in ocean chemistry on these planktonic micro-communities. Mortality assays [6, 10–12] have been used to determine the lethal dose of adverse conditions; yet, the significance of these measurements is necessarily limited as they only address the extreme conditions of planktonic life (e.g., the tolerance range to a certain chemical) [13]. For plankton species capable of actively choosing their microenvironment by cilia- or appendage-driven swimming, a more relevant measure for estimating the ecological impact of an environmental parameter such as pH and salinity should be their behavioral preference towards that parameter, often referred to as ‘preferendum’ [13, 14]. For macroscopic zooplankton, preferenda can be measured in larger tanks, where a gradient is established by diffusion through membranes or pipes in natural enclosures, layered water tanks [15–18]., and tubular Y-maze experiments [19]. However, these setups do not allow analyzing several conditions simultaneously, which would be needed to determine the preferendum. Additionally, they are lacking spatial resolution—as required for assaying different conditions within short distances, so that the planktonic organisms can sense neighboring conditions. Finally, flow velocity in the tubular Y-maze is very high and above what plankton can withstand. Only recently, a static platform with segmented agarose blocks containing different concentrations of organic compounds was devised by Zupo *et*. *al*. for testing preference of zooplankton [20]. Nonetheless this method has the limitation that the gradients generated are not stable with time. More sophisticated methods and assay systems for the quantification of plankton preferences and behavior are hence urgently needed [7].

Microfluidic technology has been applied previously to study *Drosophila* development [21], chemotaxis of cells [22], of terrestrial nematodes [23], and of marine microbes [24]; however, no effort has been made to use this technique for measuring the preferendum of zooplankton species. Here we utilize a microfluidic analysis platform that allows the quantitative study of population and individual behavior of zooplankton in response to controlled and stable gradients of ecologically relevant conditions. This is made possible by laminar flow inside the microfluidic device as a result of small channel dimensions and low Reynolds numbers. In consequence liquid streams flow parallel to each other without convective mixing, which is the working principle of our device. Smooth and stable gradients can furthermore be introduced based on the “Christmas tree” geometry [25] that chemically eliminates any step-wise profiles. We use the laminar flow device to generate co-flows of different pH streams, different algal streams and different chemo attractants and repellents in the case of predator smell experiments. Complementing this, the gradient generation device is used to generate linear salt gradients.

The minute dimensions and compactness of the microfluidic device and the possibility to monitor experiments via USB microscopes enable mobile analysis at remote locations and in the field. This further strengthens versatility and broad applicability of the microfluidic device for marine ecology research.

## Materials and Methods

### Animal culture and batches

*Platynereis dumerilii* is a marine annelid with biphasic life-cycle [26]. This meroplankton species, with broad ecological amplitude, is used as a model species in various experimental assays including ecotoxicology [27] and is known to survive at low pH/elevated CO2 conditions [28]. *Platynereis* breeding and preparation was accomplished according to the standard culturing protocol described elsewhere [26, 29]. For initiating a batch, a male and a female swarming epitokes (mature adult worms) were collected and spawned in a dish to release the eggs which were subsequently fertilized by the sperms. The dish was then maintained at 18°C and exposed to 16 h of light and 8 h of darkness to initiate embryogenesis and development. *Tetraselmis marina*, a sessile green flagellate that can be grown under bright, daylight-type artificial illumination was provided as food source during breeding. For experiments, larvae were used at 5 and 9 days post fertilization (dpf). As additional plankton species we chose a holozooplankton species of copepod: *Euterpina acutifrons*. While there has been many studies on pH tolerance in copepods [30], to our knowledge, there is nothing known about the pH tolerance specifically for *Euterpina acutifrons*. Copepods are an abundant plankton species with widespread distribution in the oceans around the world that show a different (appendage-driven) form of locomotion than *Platynereis* larvae (cilia-driven).

### Microfluidic device fabrication and experimental set-up

**Fig 1:**
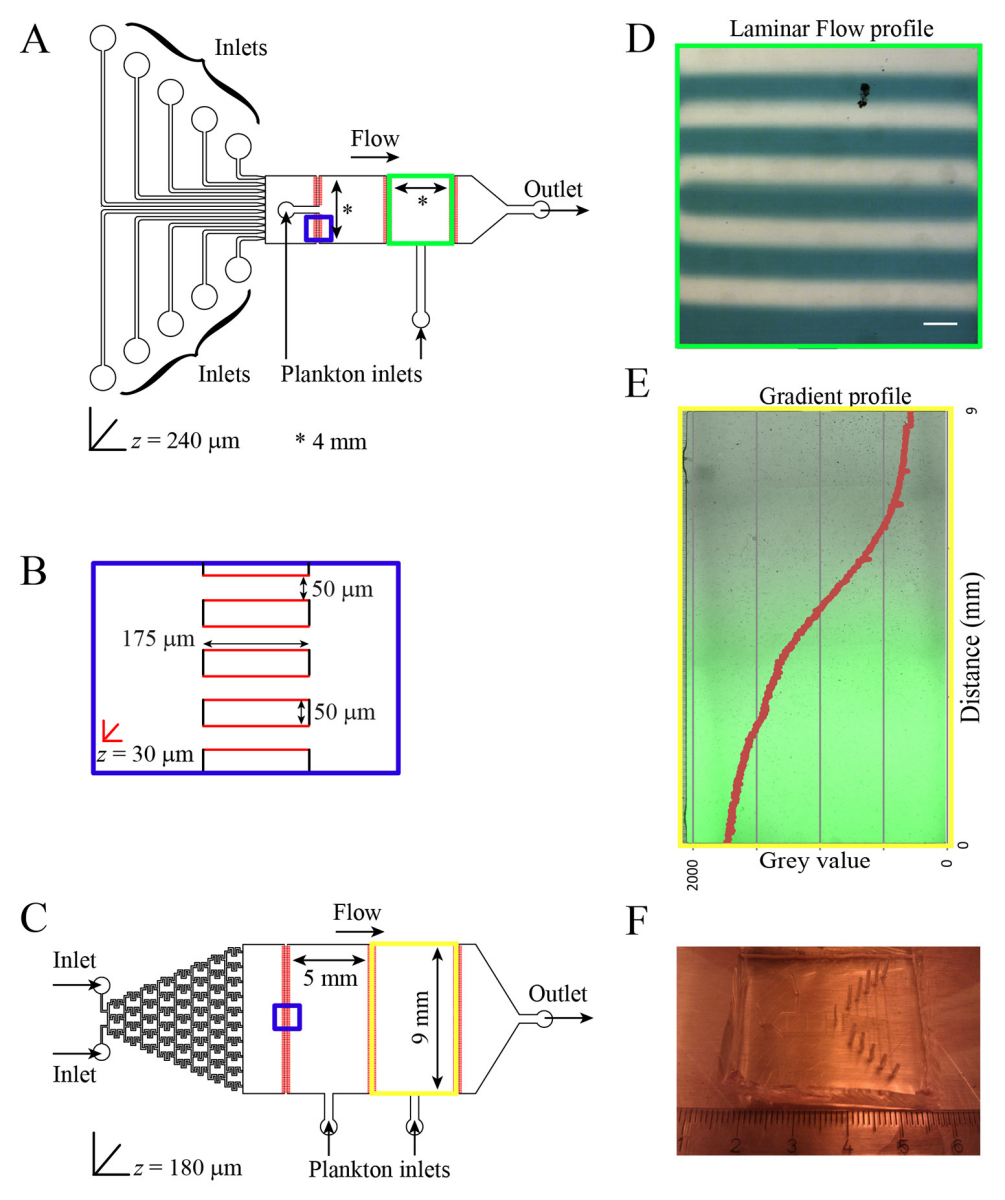
Microfluidic device geometry and flow profile. (A) Laminar flow device with ten individual inlets for different chemicals and two 4x4 mm chambers for loading plankton. (B) A magnified scheme of the shallow channels connecting adjacent chambers (blue squares in the geometries). Regions in red are manufactured to a depth of 30 μm to prevent plankton from being flushed out (C) Gradient generator device with a “Christmas tree” gradient generator and two 9x5 mm chambers for loading plankton. (D) Laminar flow as observed inside the chamber with a flow rate of 400 μl/h. A blue dye was used in every alternate stream for visualization. Scale bar represents 400 μm. (E) Gradient established within the Christmas tree gradient generator using a flow rate of 100 μl/h. Fluorescein was used to visualize and quantify the gradient. (F) A photograph of the laminar flow microfluidic device.

Multi-depth, Polydimethylsiloxane (PDMS)-based microfluidic devices were prepared using standard soft lithography [31, 32]. The mold was made using negative photoresists SU-8 2150 and SU-8 2025 (MicroChem). For our laminar flow device, firstly the SU-8 2025 photoresist was spin-coated on a silicon wafer to a depth of 30 μm and exposed to UV light passing through the photomask with the shallow connecting channels between the chambers (red in Fig 1A and 1B). Subsequently the same fabrication process was repeated over the first layer using SU-8 2150 photoresist to generate 240 μm deep structures (chambers, inlets and outlets) using a second photomask (black lines in Fig 1A). This multi-depth geometry prevents the animals from escaping the 4x4 mm chamber whilst allowing them to freely move between the different streams. The layout of the “Christmas tree” gradient generator was introduced previously [25] and manufactured as stated above (Fig 1C). The serpentine channels in the gradient generator were made 30 μm deep using a photomask also including the connecting channels between the chambers. The two chambers in this design were 9x5 mm large and had a depth of 180 μm. After the mold is prepared it can be used several times to make new devices by filling the mold with a 9:1 mix of degassed PDMS and cross-linker (Sylgard 184 silicone elastomer kit). The mixture solidifies overnight on baking at 65°C. The solidified PDMS retains the imprint of the channels in the mold and can then be cut and peeled off the mold. Access holes for inlets and outlets were punched using 1 mm biopsy punches (Harris Unicore) and the channels were closed by irreversibly bonding to glass using a plasma oven (Diener Femto).

To remove air bubbles, the device was immersed in water and degassed in a vacuum desiccator for around 15 minutes prior to all experiments. Flow rates were adjusted to 400 μl/h on each of the ten streams for obtaining laminar flow (Fig 1D) and to 100 μl/h per stream for generating a concentration gradient (Fig 1E). The plankton larvae were loaded manually using 200 μl micropipettes and closed off using needles of the appropriate size (~1 mm diameter). Tubing connection and larvae loading were all done while the device was immersed in sea water to avoid any air from entering the device.

### Image acquisition, tracking and analysis

Images were recorded using Nikon Eclipse Ti wide-field microscope (2X objective) and MotionBLITZ EoSens high speed camera from Mikrotron for experiments in the lab. Only for characterizing the gradient generator device we used a Hamamatsu ORCA 05G digital camera because this camera allowed better quantification of fluorescence. For the mobile analysis platform we used a dnt DigiMicro scale USB microscope. A screenshot freeware: Auto screen capture 2.0.5 was used to capture images every second. An experiment typically lasted ten minutes. Normally it took a couple of minutes until the laminar flow was established. The tracking of animals were done after the laminar flow was established which is visible from the dye streams. Images (around 300 frames) were cropped and pre-rotated using ImageMagick software. The tracking of moving objects was done using the motion tracker add-on by Fabian Wauthier for MATLAB 2010, using the following mixture model parameters: alpha 0.1, rho 0.01, background_thresh 0.95. Because the software tends to lose track of animals that remain at a given position for longer periods of time, a custom script was written that iteratively merges trajectories if the animal position at the ends of two trajectories was within 135μm distance. For a recording of the 300 frames (5 minutes), the average track length was 2.5 min, with at least half of the tracks going through at least 50% of the total recording time. In cases where the tracking software lost an animal (e.g., due to animals clustering together), it started a new track and the centroid positions could be determined again. This increases the number of tracks recorded per device, but usually this does not exceed 1.5 times the animal count. Using regular seawater in all ten streams, we have acquired the ‘normal’ behavioral repertoire of our animals. (Figure A in S1 File). Doing so, we defined the cumulative ‘random’ distribution of animals in the device and used this distribution as the null-hypothesis for the statistical tests of deviation. These experiments also demonstrate that there is no positional bias in the device.

### Preparation of pH, saline and algal solutions

The different pH solutions were made by adjusting the pH of sea water using HCl as an acid and NaOH as alkali base. The pH values were measured using a regular benchtop Sartorius PB11 with glass electrode pH meter. For preparing different salt concentrations, we diluted the sea water in a ratio of 1:2 with distilled water and added NaCl to obtain different concentrations (0.75x to 1.25x, 30 g/l to 50 g/l NaCl). Since the sea water is already rich in its salt content, diluting it and then adding NaCl allowed us to control the molar changes of NaCl.

We prepared microalgae extracts by filtering the algae cultures using a 0.22 μm filter and then UV treating the extract for 10 minutes to avoid algal filament formation and proliferation in long term experiments.

### Laser ablations

Ablations were done using a Zeiss FluoView 1000 cold laser. During this step, fifteen to twenty larvae were kept in 7.5% MgCl_2_ solution to impede muscle movements. A 40x objective was used and the target cells in the mouth were ablated using multiple one second laser pulses (to avoid cavitation) until the morphology (cell outlines) changed and the tissue ‘caved in’. Animals were used for microfluidic experiments on the same day.

## Results

### Tracking zooplankton behavior in a stable pH gradient

**Fig 2:**
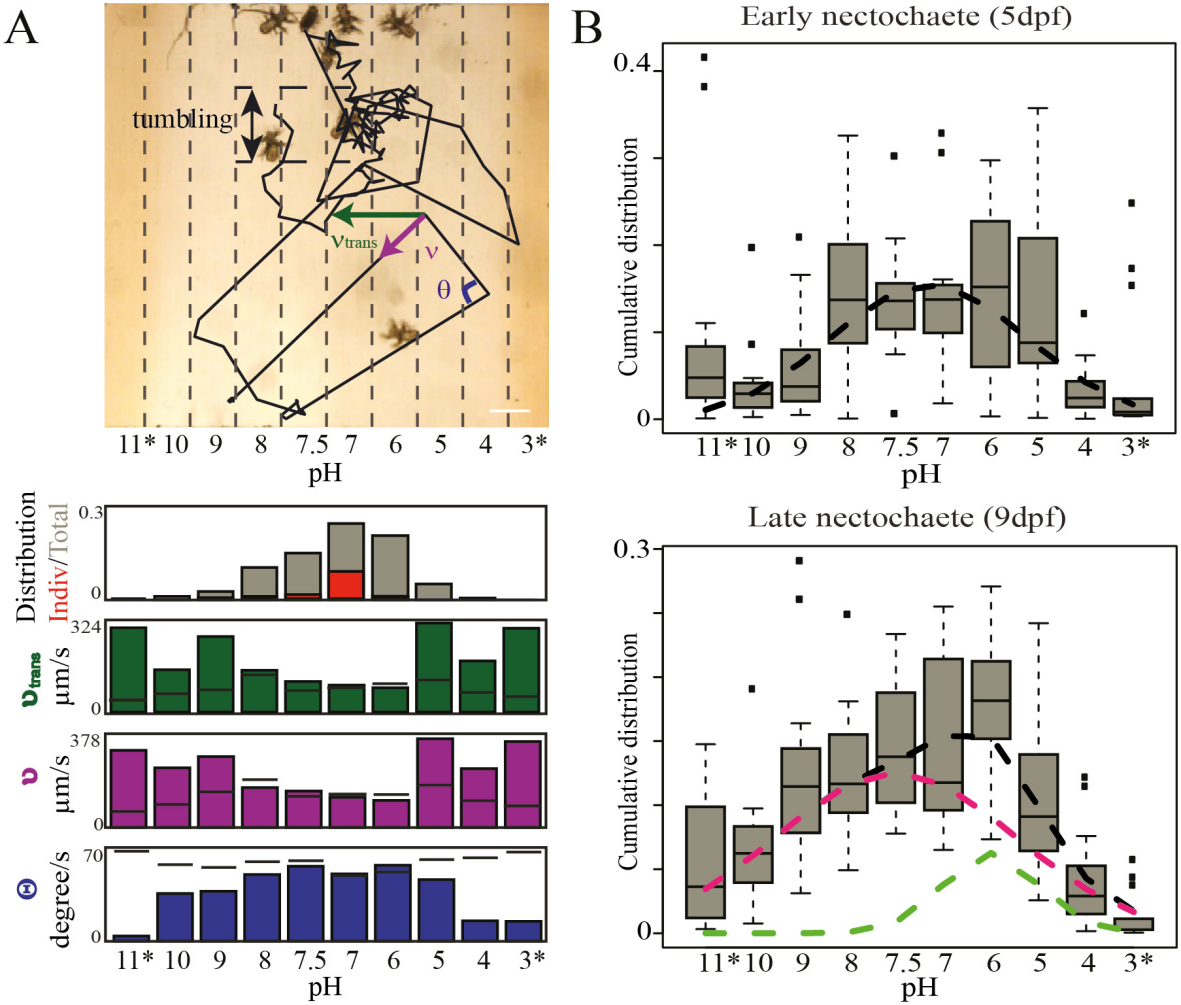
Behavioral analysis and pH preferendum of *Platynereis dumerilii*. (A) A ten-stream laminar flow microfluidic device with *Platynereis* larvae exposed to different pH conditions. Scale bar represents 400 μm. Barplots represent stream specific distribution over time for the whole population (in grey, Total) and of one example individual (in red, Indiv) as well as the stream transition speed (ν_trans_, μm/second lateral transition between streams), total speed (ν, μm/second), and change in direction or turning angle (θ, degrees/second). The black spaced lines are the control where animals were exposed to only sea water in all streams. (B) Variation in stable distributions from ten different experiments. The fitted black curves are the averages of the two distributions. Pink and light green curves are two different populations. * indicates dye stream.

We designed two microfluidic devices for our experiments: one to generate chemically distinct laminar streams that flow parallel to each other without mixing and another to generate on-device gradients of chemicals using the “Christmas tree” model [25]. In both devices the animals can freely move (swim or crawl, depending on species and developmental stage) and choose their preferred zones (S1 Movie). Using automated tracking, we determined individual and population-specific behavioral parameters such as the overall speed (ν), stream transition speed (ν_trans_), turning angle per second (θ), the average time of movement (τ_mov_), number of individuals present in a stream over time (d_(x,t)_), the overall distribution over time and the resulting stable distribution that is reached after an adaptation time (Fig 2A).

We first studied the behavior of larval stages of the marine annelid *Platynereis dumerilii* [26], a plankton model species that plays an important role in world-wide marine ecosystems and is fully amenable to various experimental approaches and molecular studies. During metamorphosis, *Platynereis* larvae switch from planktonic to benthic habitats, which represents a characteristic and major ecological transition in the marine life cycle observed in the majority of animal phyla [33]. Exposing *Platynereis* to a stable gradient of pH using our laminar flow device (Fig 2A), we observed differences in behavioral pattern depending on their location in the chamber. For example, in adverse conditions (towards the edges of the chamber), we observed up to 2.5-times higher overall and stream transition speed, with animals swimming towards the middle of the chamber (Fig 2A). In the middle of the chamber, we observed non-directional ‘tumbling’ and ‘pausing’ behavior, which results in animals remaining in the preferred streams. This tumbling behavior mirrored the enhanced rate of directional change encountered at the peak of the chemical gradient of the dinoflagellate *Oxyrrhis* [24] and may thus represent a universal planktonic behavior under favorable conditions.

### The pH preferendum: individuals and populations

Initial observations of *Platynereis* behavior suggested that the animals experienced a zone of favorable conditions in a defined interval of the pH gradient. Indeed, the stable distribution was reached on average after 10 (+/-7) (n = 10) seconds after establishment of the laminar flow and showed maximal values from pH 6 to 8 in early metamorphosing larvae (Fig 2A), which means that animals were actively avoiding undesirable (pH 5 and 9) conditions. Experiments shifting the position of pH streams were additionally performed to confirm these results and to rule out any positional bias (Figure B in S1 File). Based on the stable cumulative distribution (e.g. Fig 2B), we defined a “comfort zone”, which represents the range of conditions for a given parameter for which the presence of specimens does not significantly deviate from the maximum recorded. We propose that our comfort zone is a good comparative measure to the “preferendum”, as defined on theoretical grounds by ecologists [14]. *Platynereis* shows a relatively broad comfort zone for pH. [34]. The comfort zone is characterized by slower movement speed as well as more pronounced tumbling (which increases only when animals reach the edge of the comfort zone). Unexpectedly, for *Platynereis* larvae that have reached settlement stages (late nectochaete) our data revealed a bimodal distribution (best fitted by a mixture model of two Gaussians with RMSE of 49, compared to RMSE of 92 when fitting a single distribution, (Fig 2B, Figure C in S1 File), unraveling the presence of animals with a shifted comfort zone towards lower pH values (light green fitted curve, Fig 2B). This indicates the existence of a subpopulation of *Platynereis* settling at more acidic conditions.

### The salinity comfort zone

**Fig 3:**
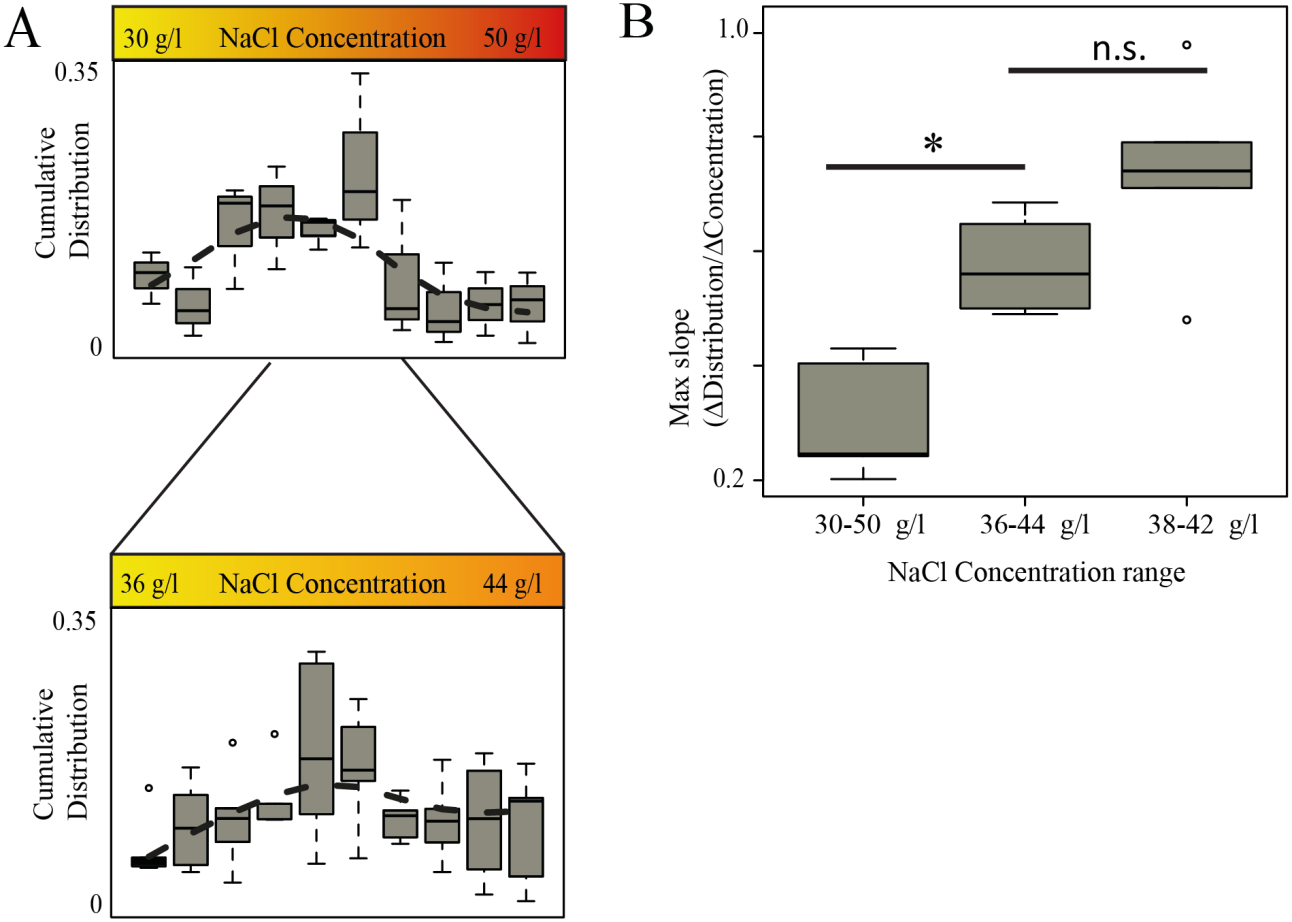
Estimating responsiveness. (A) Stable distributions at different salinity gradients from five experiments; (B) highest slope of the distribution at different ranges of salt concentrations, * = p-value is less than 0.05 (Wilcoxon rank-sumt test). n.s. = p-value is greater than 0.1

Using our gradient generator device we exposed *Platynereis* to different NaCl ranges (30–50 g/l, 36–44 g/l, 38–42 g/l etc.) and observed a preference for conditions around 34–42 g/l (Fig 3A). As for the pH gradient, we noted that the salinity comfort zone was characterized behaviorally by reduced stream transition speed and higher turning angles when compared to behavior under more adverse conditions, confirming our finding of behavioral changes in the comfort zone (Figure D in S1 File). No evidence for a ‘subpopulation’ in the stable distribution could be observed for salinity changes.

### Comparing responsiveness to pH and salinity

It is plausible that the intensity of the behavioral reaction to a given environmental parameter (such as pH or salinity) is directly related to its ecological importance [9]. We thus set out to quantify the behavioral responsiveness to a given parameter from the recorded distributions of plankton in our device, taking advantage of the different shapes of the distributions. Mathematically, the strongest change in behavior is the point with the highest slope in the stable distribution (inflection point). To find the minimal difference in molar concentration (responsiveness ‘r’, [mol/l]) for which a behavioral change can still be detected, we repeatedly zoomed into narrower concentration ranges until the slopes remained constant (Fig 3A). For salinity, this was achieved already in the 36–44 g/l range (Fig 3B) and we thus determined that *Platynereis* larvae responded to a minimal molarity change of ~13.7 mmol/l NaCl at the edges of the comfort zone. Due to the much smaller differences in the ionic concentrations on the pH gradient as compared to salinity gradient (molar), the animals were much more responsive to changes in proton concentration (response observed already at 1 μmol/l changes from the comfort zone). Interestingly, in the lower pH range the *Platynereis* response curve resembles that obtained for candidate pH receptor molecules such as acid-sensing ion channels (ASICs) [35, 36], suggesting their involvement.

### Direct comparison of different species

**Fig 4:**
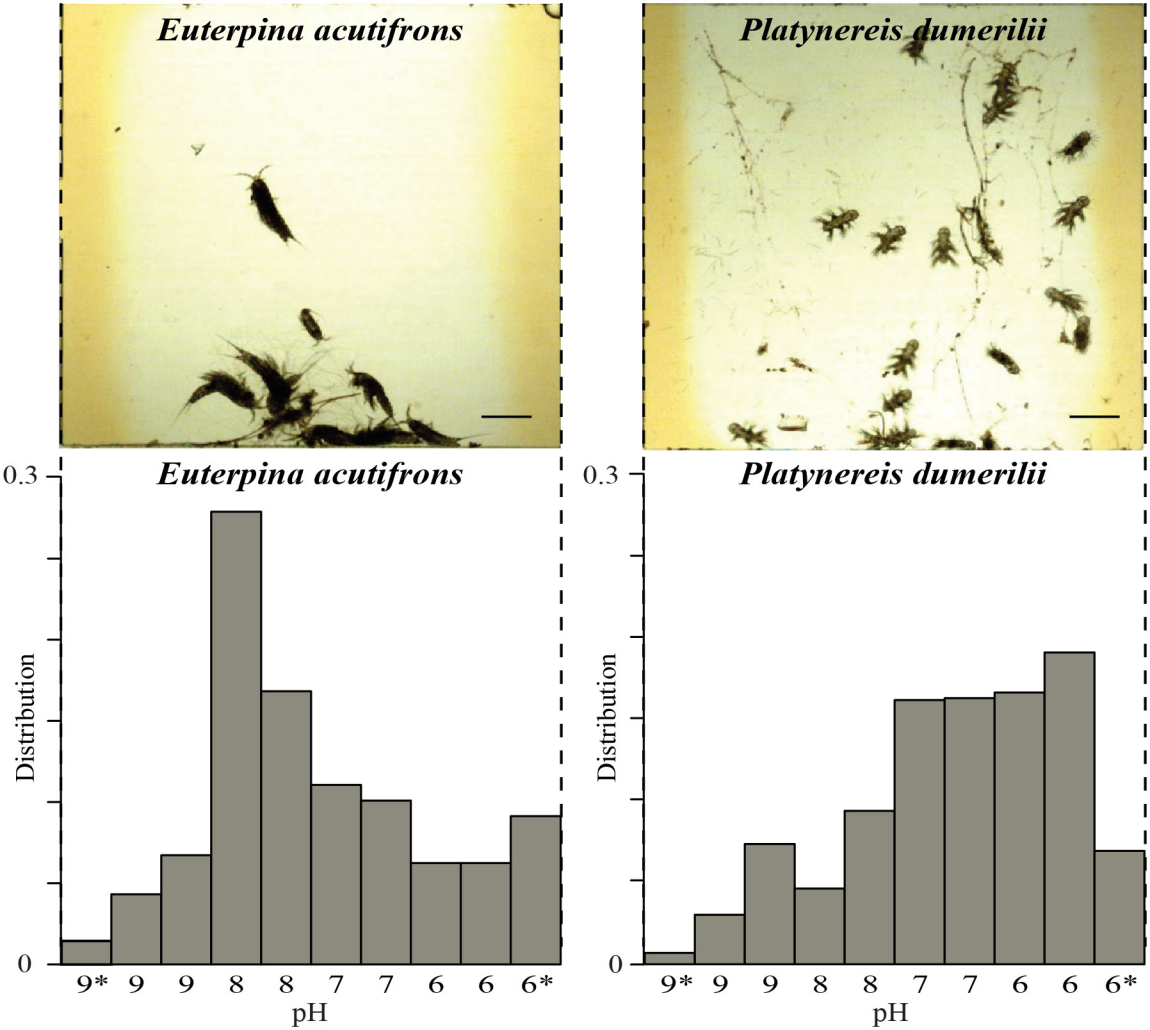
Comparing pH preferendum of *Euterpina acutifrons* and *Platynereis dumerilii*. Top panel shows the view inside two different chambers on a single device, hosting one plankton species in each. Scale bar represents 400 μm. Bar plots show the distribution. pH values are indicated on the x-axis. Two streams per pH condition were used to widen the condition and allow the larger copepods to settle in their preferred conditions. * indicates dye streams.

We also compared the pH comfort zone of another abundant planktonic copepod species collected in coastal waters (*Euterpina acutifrons*) with *Platynereis* in parallel chambers on the same device. This species, unlike *Platynereis*, showed a much narrower pH comfort zone (Fig 4). We observed that copepods in their comfort zone (between pH8 and pH9) showed similar behavioral parameters (like tumbling and transition speed) as *Platynereis* (Figure E in S1 File).

### Mobile analysis platform

Making use of the compactness of the device and the possibility to acquire images that can be tracked with a simple USB microscope (dnt DigiMicro Scale), we also performed experiments on remote sites/ships. In particular, we screened plankton preference for predator ‘smell’ and pH for animals collected at marine stations in Banyuls-sur-mer and Roscoff, France. Animals actively avoided streams with seawater taken from sea bass tanks at the aquarium in Banyuls-sur-mer (Figure F in S1 File, S2 Movie). The stable distribution was attained after 2.5 minutes taking into account the time required to establish the streams. In this experiment each stream was 4.5 mm wide. The delay in attaining the stable distribution in comparison to the pH experiments could be due to the extended stream width.

### Linking cellular function and behavior

**Fig 5:**
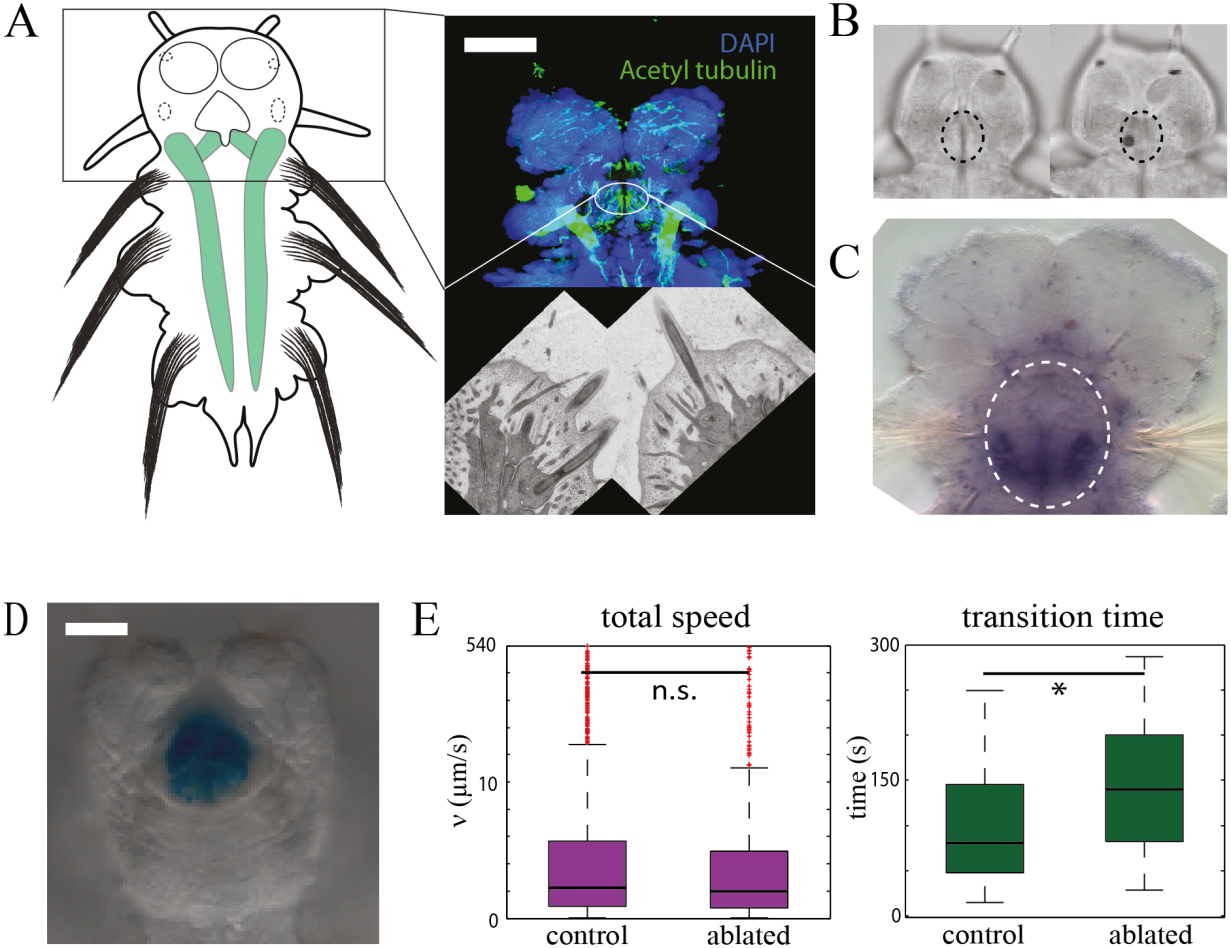
Functional characterization of cell types. (A) Location of the ciliated cells and their morphology with fluorescence and transmission electron microscopy. Scale bar represents 30 μm. (B) Ablation procedure: ciliated cells are marked by dashed circles in the 5 dpf animal (left), and are lost after ablation (right). (C) Expression of noelin in the ciliated cells of the oval demarcates foregut mouth region, indicating a putative olfactory function. (D) Ability of ciliated region to trap particles (alcian blue staining). Scale bar represents 30 μm. (E) Behavior of control and ablated animals on the microfluidic device. * = p-value 0.02 (Wilcoxon rank-sum test). n.s. = p-value greater than 0.1.

We have exposed *Platynereis* larvae to UV treated microalgal extracts of *Dunaliella* and *Isochrysis*, two of the common marine micro-algae and observed a preference towards *Dunaliella*. To explore the physiology underlying this behavior, we took advantage of our laminar flow device to experimentally determine the chemosensory cell types mediating this preference. Long ciliated cells in the ventral part of the *Platynereis* larval foregut express *noelin* [37] (Fig 5A–5C), a member of the olfactomedin family that form part of the extracellular matrix in vertebrate olfactory epithelium [38]. These cells secrete mucus (Fig 5D) for particle trapping, a common feeding mode of marine larvae [39]. Cold laser ablation of these cells doubled transition time into the *Dunaliella* stream, while the overall swimming speed remained unaffected (Fig 5E), indicating that the ciliated cells in the mouth are involved in the chemotaxis response. For these studies we ablated fifteen to twenty larvae per experiment.

## Discussion

Global environmental change, in particular ocean acidification, has started to affect marine species composition as also observed by declining coral reefs [40, 41]. It has become increasingly important to understand how individual species respond to these changes in order to identify sensitive and resilient species. This identification is necessary to predict species adaptation and how the changes in species composition will affect the ecosystem on the whole. Our study demonstrates the possibility to quantitatively measure ecological preferences of individual, actively swimming zooplankton using a microfluidic device. The advantage of using microfluidics for identifying ecological preference is the ability to generate stable gradients over time and expose a single population to multiple conditions simultaneously. Here, the response of zooplankton to ecological factors such as pH, salinity and food were analyzed.

Depending on the geographic locations, and including extreme habitats, marine pH values range from 6.6 +/- 0.5 to 8.1+/- 0.1 [42]. Our assays exceeded this range to allow measuring broad preferences. By doing so, we found that *Platynereis* larvae (5dpf) indeed displayed a broader, preferendum compared to the ambient ocean pH around 8.1+/-0.1 [43]. Such broad pH preferendum of Platynereis might relate to its broader ecological amplitudes [27, 28]. The *Platynereis* laboratory culture has originally been collected in the vicinity of acidic springs where *Platynereis* were reported to be found at very low pH [44], which might, at least in part, explain the broad comfort zone observed in our experiments. Arguably, despite known extended dial vertical migration [45] and estuary habitat, the copepod Euterpina is still adjusted to a more narrower pH range. It will be interesting to explore possible variations in the preferenda of animals populating ocean versus estuary /coastal populations.

Taking advantage of the refined and quantitative analysis of individual behavior that is possible in the microfluidic device, we identified a subpopulation within the *Platynereis* laboratory culture that consistently showed a preference to more acidic pH. This finding is important with regard to ocean acidification [46], as this subpopulation may show genetic variation that renders it more tolerant towards acidic pH. This hypothesis could be tested and validated by isolation and differential sequencing of the respective subpopulations. Since microfluidics technology has been has been successfully employed for precise phenotypic sorting [47, 48], such approach would significantly broaden the scope of our device.

Just as pH, ocean’s salinity is both dependent on the geographical location and depth [49]. The variationis ranging from 31 to 38 g/l NaCl excluding estuaries [50]. Generally, polychaetes are stenohaline (adapted to a relatively narrow salinity tolerance range), as the coelomic fluid osmolarity usually follows that of the external environment [51]. While several nereid species are reported to be tolerant of estuarine conditions through active osmoregulatory responses [52] it is generally assumed that polychaetes rather show behavioural osmoregulation, i.e., through avoiding uncomfortable conditions. Our device allows exact measurements of such preferendum. Previous reports on the impact of salinity changes on plankton were mostly based on mortality assay or hatchling rates of, e.g. copepods [53, 54], and thus focused on tolerance rather than preference. Other studies determined swimming speed in defined salinity conditions but did not provide gradients [55]. Generating density gradients by facilitating diffusion of chemicals have previously been reported and used for plankton preference measurements in larger tanks [18, 56]. Similar to this, but on a smaller scale and amenable to high-throughput imaging and tracking, our microfluidic device also allows generation of controllable and stable/well-defined gradients. Doing so, we found that *Platynereis* preferred a salinity range between 34–42 g/l, average of 38 g/l or ambient ocean salinity [43] that is also used for lab culturing. Moreover *Platynereis* required a ~13.7 mM change in NaCl concentration to elicit a response while they already responded to a 1 μM change in H^+^ ion concentration. This suggests that changes in pH probably have a more significant effect on plankton when compared to salinity changes.

In addition to physical parameters, biotic factors also influence zooplankton behavior; marine plankton mainly survive on chemical cues from their surroundings [57]. The choice of substrate however can vary significantly even in closely related locations. Initial experiments on foraging were done using microcapsules filled with different algal homogenates [58], proving that plankton can efficiently sense specific algal substrates via chemical cues. These results set the stage for extensive usage of our microfluidic device to dissect and quantify foraging behavior. For example, since our microfluidic device allows the tracking of single individuals, we used cold lasers to ablate particular cell type in *Platynereis* larvae and to track their individual response to algal extracts. A significant delay in response from the ablated individuals suggests the involvement of the ablated ciliated cells in the perception of food smell and the initiation of foraging behavior. This pioneer experiment also highlights the possibility of creating a functional cell type atlas by screening and mapping of cells detecting different sensory modalities.

Finally, inter and intra-species interactions can be systematically analyzed with our device. It is known that plankton sense their mate [59], kin [60] or predator [61] by chemical cues such as pheromones or kairomones. In a pioneer experiment, we examined the behavior of freshly collected plankton to predator smell (Sea Bass-conditioned water), monitoring a repelling behavior to the conditioned water. Here, the compactness of the device allowed conducting such experiments in remote locations such as marine stations where a huge diversity of possible interacting species can be collected and tested; and such approaches could be combined with the analysis of freshly collected plankton. Further, with the use of quantum dots and using it as a species identifier as demonstrated previously [62], it may be possible to track species interactions in a more sophisticated manner. Therefore, the applicabilty of the microfluidic device goes far beyond the applications pioneered and presented in this paper.

## Conclusions

Our microfluidic device allows determining the individual behavior of zooplankton in response to standard oceanographic parameters, promoting the study of marine ecology to a new quantitative level. In particular, by quantifying the ecological preferendum and the responsiveness to both physical and biological cues, this device extends the ‘classical’ ecological network reconstruction based on species abundance [63] by providing the information on the actual response dynamics. This provides new important data to assess network stability to both global climatic changes and local perturbations due to human activity. The microfluidic device will further allow quantifying the impact of environmental change on individual species. For example, lower pH preference as measured for *Platynereis* larvae may be advantageous in overall acidifying waters. Finally, the microfluidic device allows exposure of experimentally- or genetically-modified individuals to defined ecological conditions and thus opens up new avenues into marine molecular ecology.

## Supporting Information

S1 File **Figure A: Random distribution inside the device with all streams containing sea water**. The p-value from ANOVA test is 0.97. **Figure B: End point distribution of a pH experiment with *Platynereis***. The pH value of each stream is indicated. **Figure C: Individual level statistics of responses in early and late nectochaete larvae of *Platynereis***. A distribution with rows representing data points of the location of every individual larva over time. Red line: linear best fit to the medians; green line: linear best fit to the different response shown by a subset of larvae with broader pH tolerance range. Boxplots show average distribution with quartile range represented as rectangle. * indicates dye streams. **Figure D: Behavioral analysis of *Platynereis* in NaCl gradients**. (i) Stable distribution from five different experiments. (ii) The transition speed is higher at the edge of the preferendum. (iii) Turning angle is higher in the preferendum because of the tumbling behavior. **Figure E: Behavioural analysis of *Euterpina acutifrons* (a copepod) to pH gradients**. Turning angles (left) and transition speed (right). Note elevated turning angles in the preferendum and increased transition speed at the basic border of the preferendum. **Figure F: Analysis of freshly collected plankton preferendum at remote marine stations**. Response of *Platynereis* nectochaete to sea bass ‘smell’. The overall distribution of the experiment is shown below. Each stream was subdivided into five regions to increase the spatial resolution.
(DOCX)

S1 Movie *Platynereis* larvae (5dpf) exposed to ten different pH conditions (top to bottom: 3*,4,5,6,7,7.5,8,9,10,11*) in the laminar flow device. The flow direction is from right to left. * indicates streams with dye. The field of view covers the entire device. The video speed is 10 frames per second (fps)
(MOV)

S2 Movie Mobile analysis platform. Freshly collected *Platynereis* exposed to natural sea water (left) and water from sea bass tank (right). The field of view covers the entire device.
(AVI)
