# Supplementary material for: Quantifying Preferences and Responsiveness of Marine Zooplankton to Changing Environmental Conditions using Microfluidics
Source: PLoS One. 2015 Oct 30;10(10):e0140553. doi: 10.1371/journal.pone.0140553 (PMC4627805; doi:10.1371/journal.pone.0140553)
Supplement: S1 File — Figure A: Random distribution inside the device with all streams containing sea water. The p-value from ANOVA test is 0.97. Figure B: End point distribution of a pH experiment with Platynereis. The pH value of each stream is indicated. Figure C: Individual level statistics of responses in early and late nectochaete larvae of Platynereis. A distribution with rows representing data points of the location of every individual larva over time. Red line: linear best fit to the medians; green line: linear best fit to the different response shown by a subset of larvae with broader pH tolerance range. Boxplots show average distribution with quartile range represented as rectangle. * indicates dye streams. Figure D: Behavioral analysis of Platynereis in NaCl gradients. (i) Stable distribution from five different experiments. (ii) The transition speed is higher at the edge of the preferendum. (iii) Turning angle is higher in the preferendum because of the tumbling behavior. Figure E: Behavioural analysis of Euterpina acutifrons (a copepod) to pH gradients. Turning angles (left) and transition speed (right). Note elevated turning angles in the preferendum and increased transition speed at the basic border of the preferendum. Figure F: Analysis of freshly collected plankton preferendum at remote marine stations. Response of Platynereis nectochaete to sea bass ‘smell’. The overall distribution of the experiment is shown below. Each stream was subdivided into five regions to increase the spatial resolution. (DOCX) [file pone.0140553.s001.docx]

Supporting Information

Quantifying preferences and responsiveness of marine zooplankton to changing environmental conditions using microfluidics

Nirupama Ramanathan^1¶^, Oleg Simakov^1,2¶^, Christoph A. Merten^1^*, Detlev Arendt^1^*

^1^European Molecular Biology Laboratory, Heidelberg, Germany

^2^Okinawa Institute of Science and Technology, Okinawa, Japan

***** Corresponding authors

E-mail: [arendt@embl.de](mailto:arendt@embl.de) (DA), [christoph.merten@embl.de](mailto:christoph.merten@embl.de) (CAM)

^¶^ These authors contributed equally to this work.

4

0

Stream 8

Stream 2

Stream 3 13

Stream 9 91

Stream 7

Stream 6 62

Stream 4

Stream 5 51

Stream 10*

Stream 1*

S1 File. Figure A: Random distribution inside the device with all streams containing sea water. The p-value from ANOVA test is 0.97. Figure B: End point distribution of a pH experiment with *Platynereis.* The pH value of each stream is indicated. Figure C: Individual level statistics of responses in early and late nectochaete larvae of *Platynereis*. A distribution with rows representing data points of the location of every individual larva over time. Red line: linear best fit to the medians; green line: linear best fit to the different response shown by a subset of larvae with broader pH tolerance range. Boxplots show average distribution with quartile range represented as rectangle. * indicates dye streams. Figure D: Behavioral analysis of *Platynereis* in NaCl gradients. (i) Stable distribution from five different experiments. (ii) The transition speed is higher at the edge of the preferendum. (iii) Turning angle is higher in the preferendum because of the tumbling behavior. Figure E: Behavioural analysis of *Euterpina acutifrons* (a copepod) to pH gradients. Turning angles (left) and transition speed (right). Note elevated turning angles in the preferendum and increased transition speed at the basic border of the preferendum. Figure F: Analysis of freshly collected plankton preferendum at remote marine stations. Response of *Platynereis* nectochaete to sea bass ‘smell’. The overall distribution of the experiment is shown below. Each stream was subdivided into five regions to increase the spatial resolution.

**S1 Movie:** *Platynereis* larvae (5dpf) exposed to ten different pH conditions (top to bottom: 3*,4,5,6,7,7.5,8,9,10,11*) in the laminar flow device. The flow direction is from right to left. * indicates streams with dye. The field of view covers the entire device. The video speed is 10 frames per second (fps)

**S2 Movie:** Mobile analysis platform. Freshly collected *Platynereis* exposed to natural sea water (left) and water from sea bass tank (right). The field of view covers the entire device.

Figure A. Random distribution inside the device with all streams containing sea water.


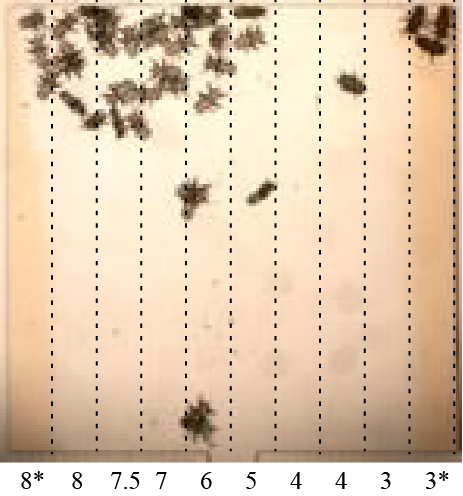


Figure B. End point distribution of a pH experiment with *Platynereis.*


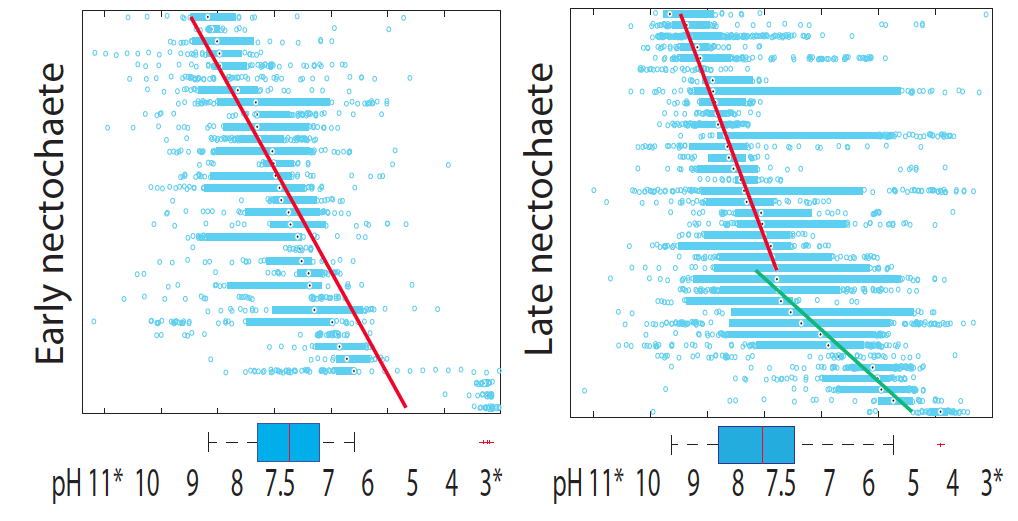


Figure C. Individual level statistics of responses in early and late nectochaete larvae of *Platynereis*.


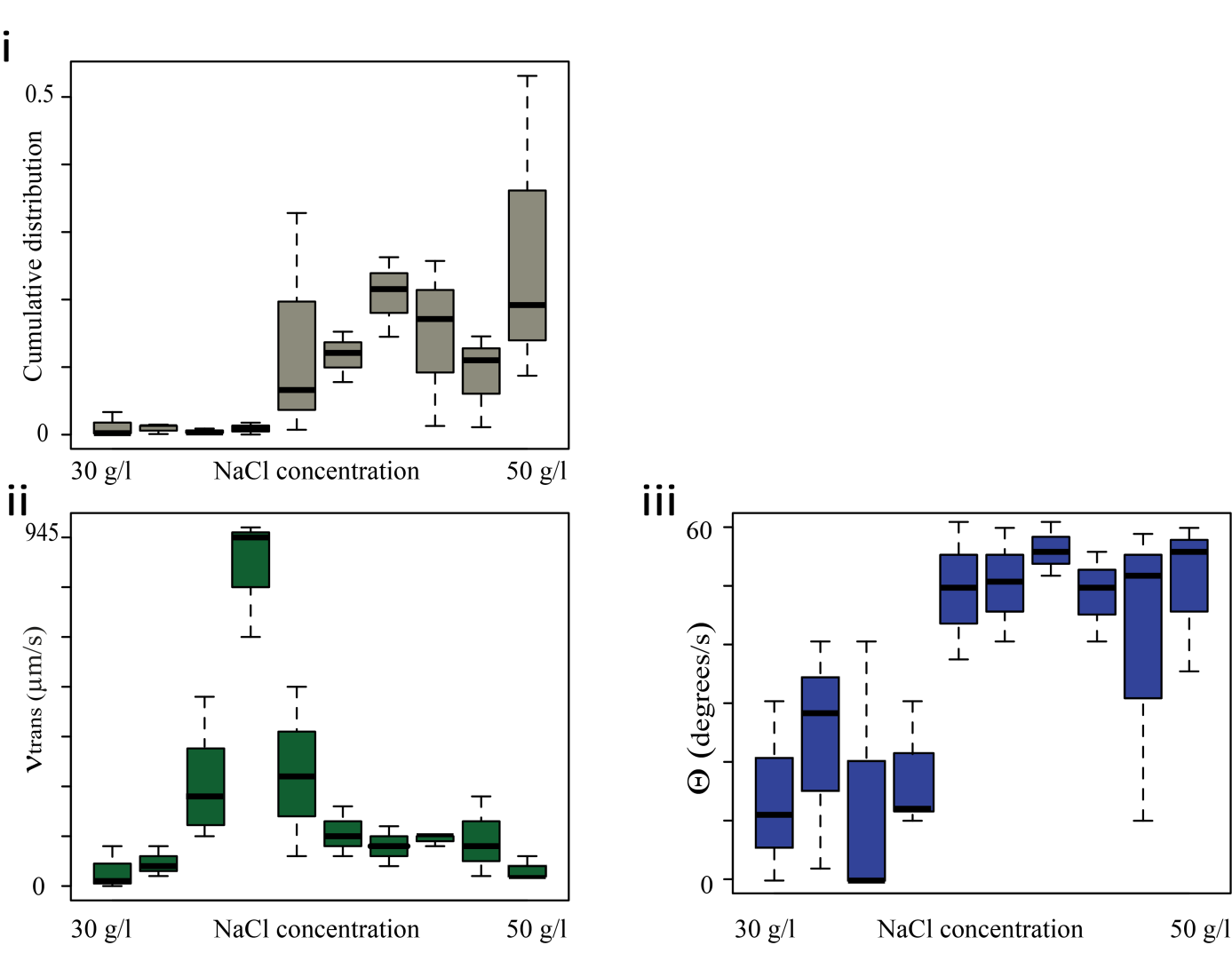


Figure D. Behavioral analysis of *Platynereis* in NaCl gradients.


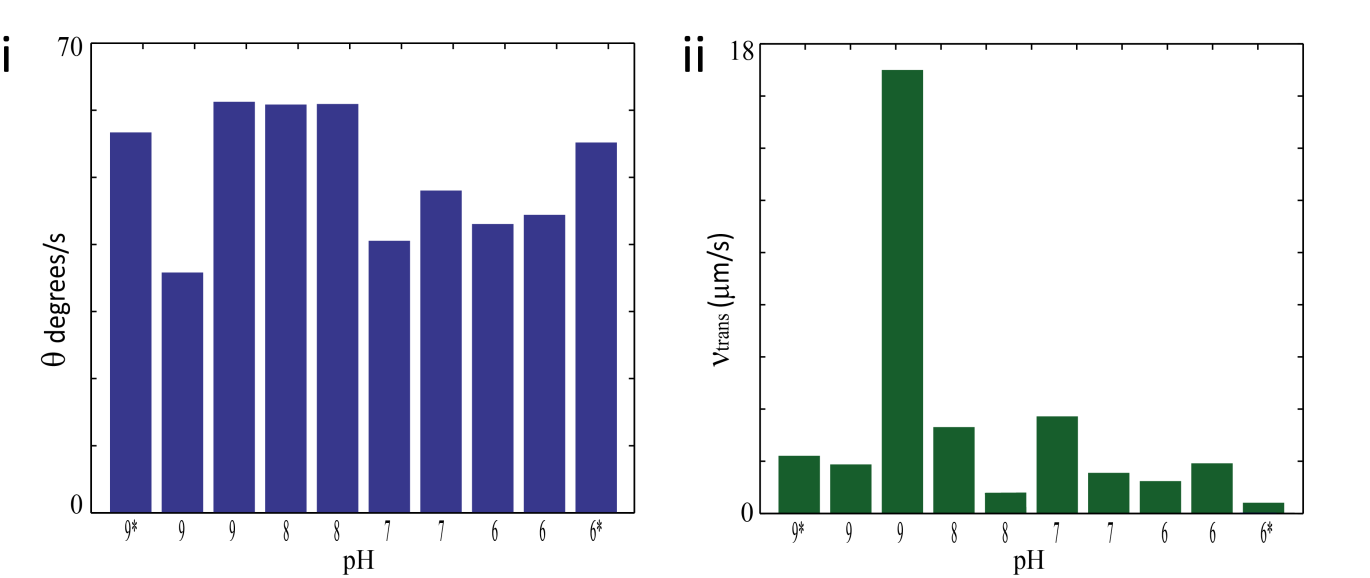


Figure E. Behavioural analysis of *Euterpina acutifrons* (a copepod) to pH gradients.

**Natural Sea Water**

**Water from tank containing sea bass**


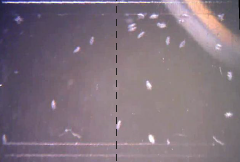


**Start**


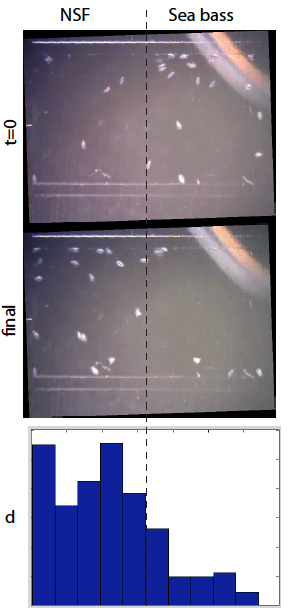


**End**


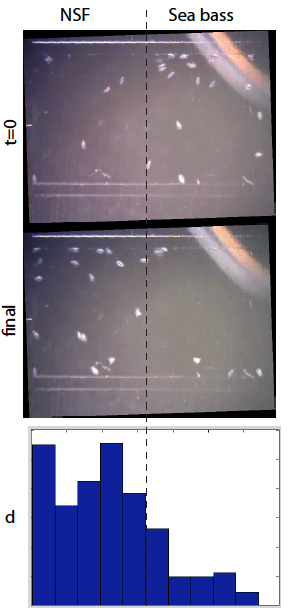


0

9

Distribution over time

Distance (mm)

Figure F. Analysis of freshly collected plankton preferendum at remote marine stations.
